# Co-existing Paraganglioma, Cholesteatoma, and Otomastoiditis With Overlapping Imaging Features: A Diagnostic Challenge

**DOI:** 10.7759/cureus.42373

**Published:** 2023-07-24

**Authors:** Tom Mishael, Babu Philip, Arun George, Sandeep S

**Affiliations:** 1 Radiology, St. John's Medical College Hospital, Bangalore, IND

**Keywords:** hrct, mri, csom, otomastoiditis, cholesteatoma, paraganglioma

## Abstract

Head and neck paragangliomas are rare neuroendocrine tumors arising from the autonomic nervous system. Imaging hallmarks of paragangliomas of the head and neck include an enhancing soft-tissue mass in the carotid space, jugular foramen, or tympanic cavity on computed tomography; a salt-and-pepper appearance on standard spin-echo magnetic resonance imaging; and an intense blush on angiography. Imaging studies depict the location and extent of tumor involvement, help determine the surgical approach, and predict operative morbidity and mortality. However, an atypical presentation of paragangliomas, especially when co-existing with other middle ear pathologies that have overlapping imaging findings, can often be misleading. Here, we report a case of simultaneous occurrence of paraganglioma, cholesteatoma, and otomastoiditis in a young adult female.

## Introduction

Paragangliomas of the head and neck are rare neuroendocrine tumors arising from the extra-adrenal paraganglia of the autonomic nervous system. These tumors are commonly found near vascular structures and are typically named after their location of origin. The middle ear is the second most common site for head and neck paragangliomas after the carotid body. Jugular tumors located in the region of the jugular foramen often extend through the skull base, occurring both intra- and extra-cranially, as well as within the jugular vein. Jugulotympanic paragangliomas may exhibit a deeper red coloring when observed behind the overlying tympanic membrane [1].

Diagnosis is typically made through a combination of clinical findings and radiographic studies. Magnetic resonance imaging (MRI) is the most important imaging modality for evaluating and characterizing suspected head and neck paragangliomas. Definitive management of these lesions should be carefully considered, taking into account both tumor and patient-oriented factors, especially in terms of potential treatment-related morbidity.

Surgery and radiation therapy are the primary treatment modalities for paraganglioma. The choice of treatment depends on the tumor's size, location, biological activity, and the overall health of the patient. Although radiotherapy may effectively arrest the tumor's growth, complete elimination rarely occurs without surgical resection. Surgery, however, may be associated with significant morbidity, mainly due to the risk of major cranial nerve injury. Thus, patient selection, considering age and medical condition, should be thoroughly assessed before recommending aggressive surgery for head and neck paragangliomas, particularly for patients at risk of experiencing disabling surgical morbidity [2].

Diagnostic imaging can be considered in two clinical situations: in patients who present with clinical symptoms suggestive of a paraganglioma and in individuals from families with hereditary paragangliomas. It is necessary not only to detect and characterize the lesion but also to study the presence of multiplicity. For these purposes, MRI, especially 3D time-of-flight magnetic resonance angiography (3D TOF MRA), is the modality of choice. On imaging, paragangliomas appear as highly vascular tumors showing intense post-contrast enhancement, which can resemble tumors of vascular origin, middle ear adenoma, schwannoma, meningioma, or even chronic otomastoiditis. CT scanning is useful in demonstrating destruction of the temporal bone. Angiography, in combination with embolization, is mainly used prior to surgical resection but can also be employed for diagnostic purposes when the diagnosis is not yet clear. Several parameters play a role in the decision to treat, of which multifocality and impairment of cranial nerves are the most important. The primary therapeutic option for paragangliomas is complete excision of the tumor with preservation of vital neurovascular structures. However, resection should be balanced against a more conservative "wait and scan" policy or palliative treatments such as radiotherapy [3,4].

We report a patient with middle ear paraganglioma mimicking as well as concealed by chronic otomastoiditis and cholesteatoma, primarily focusing on radiological imaging.

## Case presentation

A 28-year-old was admitted to the Department of Otolaryngology with a one-year history of right-sided earache and hearing disturbance. The patient also complained of on-and-off pus discharge from the right ear during the same period. The hearing loss had progressively increased over the past year, and the earache had worsened in the past week. There was no history of facial deviation, dysphagia, or hoarseness of voice. Physical examination showed no palatal asymmetry or deviation of facial muscles, tongue, or uvula. Additionally, there was no significant cervical lymphadenopathy.

Otoscopic examination showed a bulging right tympanic membrane with no definitive evidence of tympanic membrane perforation. However, the tympanic membrane was sub-optimally evaluated due to thick crusts within the external auditory canal (EAC). Indirect laryngoscopy was unremarkable and showed no evidence of vocal cord palsy. Audiometry revealed a conductive type of deafness. As the patient did not show improvement with conservative management, the treating team requested a high-resolution computed tomography (HRCT) of the temporal bone.

**Figure 1:**
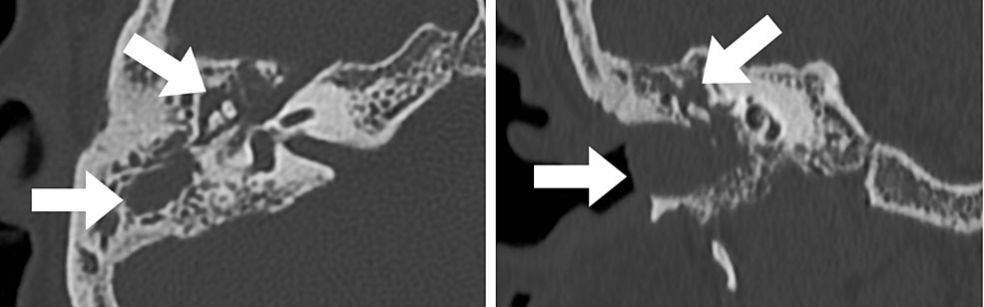
Axial and coronal reconstructed HRCT images of the right temporal bone show soft tissue thickening in the right middle ear cavity and mastoid air cells associated with bony erosions. HRCT: high-resolution computed tomography.

On HRCT of the temporal bone, we observed soft tissue thickening that obliterates the right middle ear cavity, including Prussak's space, and extends to the right mastoid cavity via the aditus (Figure 1). Additionally, soft tissue thickening was noted to involve the bony EAC, causing destruction of its anterior wall. The imaging also revealed erosions and destruction of the right posterior semicircular canal, carotid canal, sinus plate, tegmen tympani, and bony canal of the facial nerve. Furthermore, there were erosions and destruction of the posterior wall of the right mastoid antrum, with fluid and soft tissue density foci within the right mastoid air cells. The ossicular chain was intact, and the cochlea, vestibule, and other semicircular canals were normal. However, mild bony rarefaction and erosion of the ear ossicles were noted. The internal auditory canal appeared normal.

Based on the imaging findings on HRCT temporal bone, a provisional diagnosis of chronic otomastoiditis with cholesteatoma was made. However, due to the extensive soft tissue thickening and bony erosions, a contrast-enhanced MRI of the brain and skull base was suggested to look for intracranial, vascular, and other soft tissue extensions.

**Figure 2:**
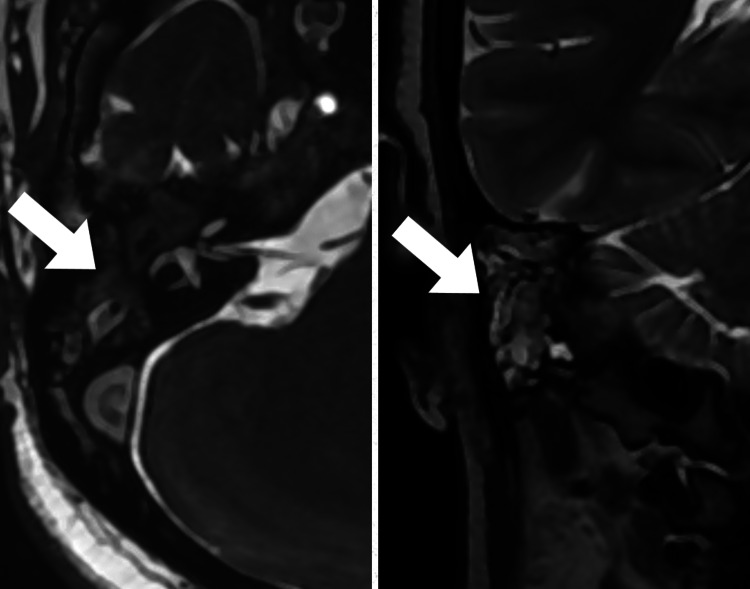
Axial and Coronal T2-weighted MR images show heterogeneously hyperintense foci within the right middle ear cavity and mastoid air cells. MR: magnetic resonance.

**Figure 3:**
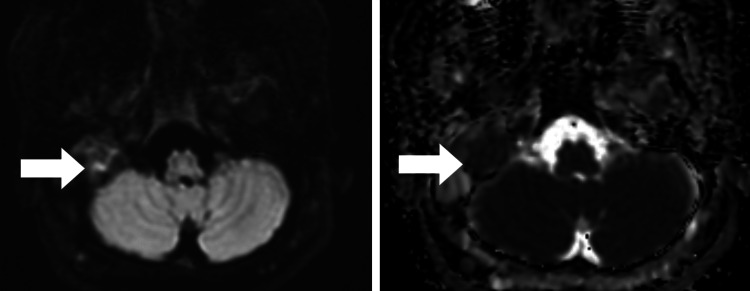
An axial diffusion-weighted MR image showing a few areas of diffusion hyperintensity in the right middle ear cavity and mastoid air cells associated with a signal drop on the ADC images. ADC: apparent diffusion coefficient, MR: magnetic resonance.

**Figure 4:**
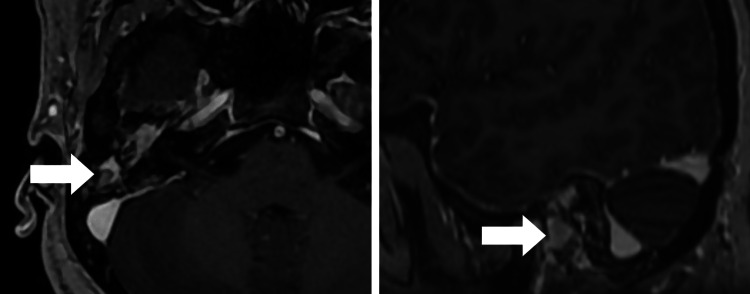
Axial and Sagittal T1-weighted post-contrast MR images showing heterogeneously enhancing components in the right middle ear cavity and mastoid air cells with a few non-enhancing and hyper-enhancing areas. MR: magnetic resonance.

MRI showed ill-defined, heterogeneously hyperintense foci on T2-weighted images in the right middle ear cavity and mastoid air cells (Figure 2), with areas of diffusion restriction (Figure 3). Corresponding areas demonstrated heterogeneous enhancement with a few non-enhancing and hyper-enhancing regions on post-contrast T1-weighted images (Figure 4). Mass effect on the right sigmoid sinus, internal jugular vein, and internal carotid artery was noted, associated with a mild focal reduction in the caliber of the right internal carotid artery. However, contrast opacification was maintained within the aforementioned vessels. There was no evidence of any intracranial extension.

A radiological diagnosis of chronic otomastoiditis with cholesteatoma was made in correlation with the contrast-enhanced MRI, HRCT, and clinical findings. However, a remote possibility of an underlying hyper-enhancing/vascular lesion was raised in view of a few hyper-enhancing areas in the middle ear and mass effect on the sigmoid sinus, internal jugular vein, and internal carotid artery on the right side.

Given the atypical imaging findings, suspicion of a hyper-enhancing space-occupying lesion in the middle ear, and the lack of improvement with conservative management, the treating team decided to proceed with surgical exploration. Intraoperatively, they found an ill-defined, irregular, lobulated lesion with a tendency to bleed in the middle ear cavity, along with features of chronic otomastoiditis and cholesteatoma in the background. The lesion was surgically excised and sent for histological diagnosis.

**Figure 5:**
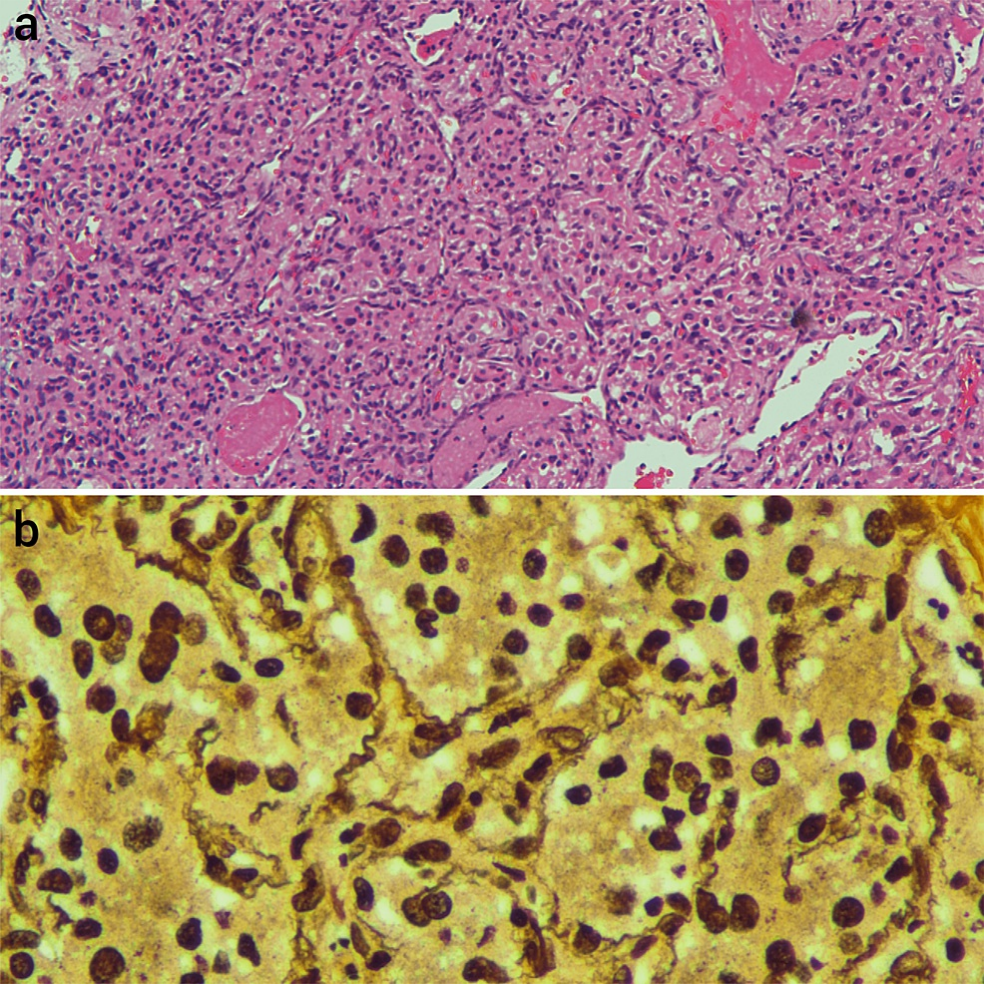
Histopathological examination using hematoxylin and eosin staining (a) and reticulin staining (b) techniques showing features of paraganglioma.

**Figure 6:**
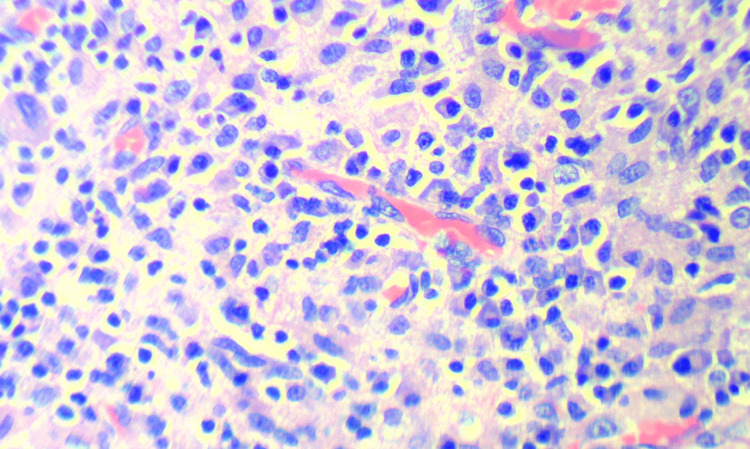
Histopathological examination using hematoxylin and eosin staining technique showing features of cholesteatoma.

Histopathological examination of the lobulated lesion was performed using hematoxylin and eosin staining and reticulin staining techniques. It revealed cells arranged in an organoid pattern with numerous blood vessels lined by normal endothelial cells. The cells were round to oval, with a few spindled forms, vesicular nuclei, prominent nucleoli, and eosinophilic granular cytoplasm. Most of the stroma appeared focally hyalinized and myxoid. There was no evidence of necrosis or increased mitotic activity. These features were consistent with jugulotympanic paraganglioma (Figure 5). No other special staining techniques were performed for the specimen. The second specimen, taken from areas of bony erosion, showed fragments of fibro-collagenous tissue with dense lymphoplasmacytic infiltrates admixed with foamy histiocytes, cholesterol clefts, foreign-body-type giant cells, keratin flakes, and bony spicules, suggestive of cholesteatoma (Figure 6). Interspersed areas of proliferation of fibroblasts, endothelial cells, thin-walled capillaries, and inflammatory cells were also found, which was suggestive of granulation tissue. 

The patient tolerated the surgery well and did not experience blood pressure fluctuations in the perioperative period. Postoperatively, the patient was monitored for complications in the ward until postoperative day 5 and was then discharged. On follow-up, the patient exhibited symptomatic improvement, with the right-sided earache resolved. Repeat audiometry three weeks after the surgery showed a significant improvement in the hearing ability of the right ear.

## Discussion

Glomus jugulare tumors are the most common tumors of the middle ear, while jugulotympanic paragangliomas are the second most common tumors of the temporal bone. There is no clear racial predilection, but females are four to six times more commonly affected than males. Glomus tumors exhibit a familial tendency, with males being more commonly involved in this form. An autosomal dominant inheritance pattern has been suggested [5].

In recent years, MRI has been used to detect and localize paragangliomas. These tumors are isointense to the surrounding structures on unenhanced T1-weighted images, making them difficult to recognize when small. On T2-weighted images, paragangliomas show increased signal intensity. Additionally, multiple punctate and serpentine areas of signal void caused by high-velocity flow in tumor vessels can be present.

Intravenous administration of MR contrast media has been recommended to improve tumor detection in patients with paraganglioma. Some studies have suggested obtaining T1-weighted images with gadopentetate dimeglumine in suspicious patients when no paragangliomas are seen on unenhanced T1-weighted images, without the addition of T2-weighted series. Although relatively safe, intravenous administration of these contrast agents is invasive, and a number of side effects, including anaphylactic reactions, can occur. Furthermore, their use increases costs and can prolong examination time. Therefore, it is essential to assess whether paragangliomas are detected significantly better when gadopentetate dimeglumine is used [6].

The overwhelming majority of head and neck paragangliomas are benign. Malignant behavior has been noted in approximately 4% of jugulotympanic tumors and 6% and 16% of carotid body and vagal paragangliomas, respectively. Histological criteria are not accepted as a definite sign of malignancy. The only acceptable criterion of malignancy is the presence of metastasis to the cervical lymph nodes or distant sites, such as the lung, liver, and skin. It is important to note that the aggressive behavior of a paraganglioma does not necessarily imply malignancy. Malignancy in paragangliomas is more common in sporadic, non-familial patients than in the hereditary form [7].

The coexistence of paraganglioma and cholesteatoma is a rare entity in itself. A definitive radiological diagnosis becomes even more challenging when there is also a superimposed otomastoiditis. In this patient, the typical imaging features of paraganglioma, such as the 'light bulb' appearance on T2-weighted images or the 'salt and pepper' appearance on T2-weighted images or post-contrast T1-weighted images, were lacking [8,9]. Additionally, there was no evidence of any well-defined lesion with intense homogenous enhancement in the middle ear. The bony erosions in the right temporal bone could have been secondary to either cholesteatoma, paraganglioma, or both, while the enhancing components in the middle ear and mastoid air cells may be attributed to either paraganglioma or granulation tissue of chronic otomastoiditis, or both [10]. The treating team must be notified about the possibility of an additional occult hyper-enhancing lesion to avoid catastrophic intra-operative incidents.

## Conclusions

HRCT is an excellent technique for demonstrating even small abnormalities of the thin and complex bony structures of the middle ear. Established indications encompass complex conditions, such as the complications of acute and chronic otomastoiditis, the postoperative ear in chronic otomastoiditis, or the localization of prosthetic devices, and the assessment of congenital or vascular anomalies. High-resolution CT precisely shows the extent of bone erosion associated with cholesteatoma. On the other hand, MRI is indicated when complicated inflammatory lesions are suspected to extend into the inner ear or towards the sigmoid sinus or jugular vein. It is also valuable in cases where fistulization through the tegmen tympani or the posterior wall of the temporal bone is usually detectable by CT, but the actual involvement of meninges and veins requires better assessment.

Neoplasms arising from or extending into the middle ear necessitate the use of both HRCT and MRI, as their combined data provide essential information for surgical planning, particularly regarding the destruction of thin bony structures and the relationships of the lesion with the dura and surrounding vessels. Furthermore, digital subtraction angiography and interventional vascular techniques play an essential role in the pre-surgical work-up and embolization of paragangliomas extended into the middle ear.

Head and neck paragangliomas may not always present as intensely enhancing T2 hyperintense, polypoidal lesions with flow voids on MRI. Overlapping imaging features and atypical clinical presentations can be misleading, leading to diagnostic dilemmas. When there is even the slightest suspicion of a neoplastic lesion, especially a highly vascular one for which biopsy is contraindicated, the radiology team must forewarn the surgeons. Both radiologists and clinicians should be aware of atypical imaging findings and their confounding factors, and they should diligently look for possible co-existing pathologies and ancillary findings while evaluating middle ear pathologies, even if a diagnosis that explains the patient's symptomatology has already been obtained.
